# Weekly, Seasonal, and Geographic Patterns in Health Contemplations About Sundown Syndrome: An Ecological Correlational Study

**DOI:** 10.2196/13302

**Published:** 2019-05-28

**Authors:** Kenneth Michael Madden, Boris Feldman

**Affiliations:** 1 Gerontology and Diabetes Research Laboratory University of British Columbia Vancouver, BC Canada; 2 Centre for Hip Health and Mobility University of British Columbia Vancouver, BC Canada

**Keywords:** sundown syndrome, geriatric medicine, dementia, circadian rhythms, infodemiology, infoveillance, internet

## Abstract

**Background:**

Sundown syndrome (ie, agitation later in the day) is common in older adults with dementia. The underlying etiology for these behaviors is unclear. Possibilities include increased caregiver fatigue at the end of the day and disruption of circadian rhythms by both age and neurodegenerative illness.

**Objective:**

This study sought to examine circumseptan (weekly) patterns in search volumes related to sundown syndrome, in order to determine if such searches peaked at the end of the weekend, a time when caregiver supports are least available. We also sought to examine both seasonal differences and associations of state-by-state search activity with both state latitude and yearly sun exposure.

**Methods:**

Daily Internet search query data was obtained from Google Trends (2005-2017 inclusive). Circumseptan patterns were determined by wavelet analysis, and seasonality was determined by the difference in search volumes between winter (December, January, and February) and summer (June, July, and August) months. Geographic associations between percent sunny days and latitude were done on a state-by-state basis.

**Results:**

“Sundowning” searches showed a significant increase at the end of the weekend with activity being 10.9% (SD 4.0) higher on Sunday as compared to the rest of the week. Search activity showed a seasonal pattern with search activity significantly highest in the winter months (36.6 [SD 0.6] vs 13.7 [SD 0.2], *P*<.001). State-by-state variations in “sundowning” searches showed a significant negative association with increasing mean daily sunlight (*R*^2^=.16, β=-.429 [SD .149], *P*=.006) and showed a positive association with increasing latitude (*R*^2^=.38, β=.648 [SD .122], *P*<.001).

**Conclusions:**

Interest in “sundowning” is highest after a weekend, which is a time when external caregiver support is reduced. Searches related to sundown syndrome also were highest in winter, in states with less sun, and in states at more northerly latitudes, supporting disrupted circadian rhythms as another contributing factor to these behaviors.

## Introduction

Agitation and aggression in older adults with dementia are accompanied by significant emotional costs for caregivers and accounts for approximately 30% of the total annual cost of caring for a person with Alzheimer’s disease in the community [1]. One of the most troublesome and common behavioral issues is the “sundown syndrome,” which is characterized by increasing agitation, confusion, and anxiety later in the day (late afternoon or evening) [2]. Sundown syndrome has shown to be present in 66% of community-dwelling older adults with dementia and greatly increases the risk of institutionalization [3].

The term “sundowning” was first used in the scientific literature by Cameron et al in 1941 when he described an increase in disorientation and agitation in a dementia patient placed in a darkened room [4]. The first systematic look at sundown syndrome found that 11 out of 89 facility patients exhibited these behaviors and that they were related to environmental factors such as the smell of urine, being awakened frequently, or being new to the facility as opposed to physiological ones [5]. Exum et al looked at the use of “as needed” (prn) medications in institutionalized older adults and found that medication use to control difficult behaviors occurred at institutionally defined times such as shift change (reflecting caregiver fatigue) as opposed to changes in ambient light [6].

Despite the large prevalence of caregiver reports of temporal changes in behavior, there is a lack of detail with respect to the underlying mechanisms [7]. Some work has suggested that there is no biological mechanism underlying behavioral issues in the late afternoon/early evening and is merely due to the effects of caregiver fatigue on subjective impressions [8]. Other work, however, has shown temporal increases in agitation and anxiety in animal models [7] and in human observational studies [9]. Due to the well-established impact of neurodegenerative dementias on circadian rhythms [10], some early studies have shown beneficial effects of both light therapy [11,12], increased natural light [13], and melatonin [14].

Traditionally, behavioral issues in dementia have been measured using clinical scales [15] and surveys [16]. One drawback to using these methods to determine temporal and geographic patterns is the long time lag between measurement and analysis [17] and the tendency of persons to answer in a socially desirable manner [18]. A newer technique, referred to as “infodemiology,” allows researchers to examine the hidden concerns and motivations of large populations using open access Internet search activity [19]. Open access Google search data have allowed us to find patterns in various populations’ hidden health concerns on a real-time basis. For example, recent work has determined which day of the week people contemplate smoking cessation [20] and which day of the week is the “healthiest day” [21]. Internet search data have also been used to find seasonal and geographic patterns in contemplations surrounding weight loss [22], exercise [22], restless legs syndrome [23], and mental health [24].

The current study uses United States Google search data to explore both caregiver fatigue and disrupted circadian rhythms as underlying mechanisms for sundowning behaviors. If caregiver fatigue is a factor in the interpretation of the behavior of older adults, we hypothesized that Internet search activity should peak at the end of each weekend, a time when caregiver supports are least available [25]. If alterations in circadian rhythms are a factor, search activity should be higher during winter months and state-by-state search activity should show positive associations with both increasing state latitude and decreasing sun exposure.

## Methods

### Internet Search Activity

The number of searches that have been performed for any given keyword can be computed using Google Trends, a Web-based tool. Since overall search activity often varies on different days of the week (eg, search activity is different on weekend vs non-weekend days), search is normalized for the overall number of searches and is reported as a score between 0 and 100 [26]. This normalization of search activity avoids biases due to changes in search activity (eg, during the winter vs summer months, or on weekend days) [26]. Search activity can also be narrowed to a specific country or state within a country. As per current standards for reporting Google Trends data in medical studies [27], daily search data were obtained from 2005-2017, and the database was downloaded as a .csv file accessed on September 7, 2018. The complete text for all queries was “sundowning.” All searches were limited to those classified by Google as in the “Health” subcategory in order to avoid non–health-related searches. As in previous studies of this type [21,27], this paper used only open access, publicly available aggregate data. A human subjects ethics board review was deemed unnecessary by our institution [28].

### Circumseptan Temporal Pattern Analysis

The circumseptan (weekly) periodicity in “sundowning” searches for 2016 was determined using a continuous wavelet transformation [29], using the WaveletComp package in R version 3.4.2 [29]. Continuous wavelet transformations are similar to other methods of determining periodicity in time series analysis, such as cosinor or Fourier transformation analyses. The advantage of the wavelet transformation analysis is that there are no parametric assumptions required. Internet search data can often show long-term trending bias that can obscure short-term periodicities when other methods are used. Wavelet transformations are robust in the face of bias and allow us to detect more short-term patterns such as seasonal or circumseptan variations. It has also been used in previous studies to determine weekly patterns of health contemplations [20,21]. The time series was reconstructed with all periodic components less than 14 days after adding back the mean of the time series (wavelet transformations are centered about the mean), as in previous studies [20,21].

### Seasonal Temporal Pattern Analysis

The magnitude of the seasonal shifts in search inquiries for “sundowning” was determined by the difference between the average volume of searches in winter months (December, January, and February) and summer months (June, July, and August) as done in previous studies [22,24]. Seasonal analysis was performed for all data from 2005-2017.

### Latitude and Natural Light Exposure Data

Latitudes for the center of each state were obtained from the US Department of Commerce [30], and average percent daily hours of sunshine data for each state were obtained from the National Centers for Environmental Information [31] for 2016.

### Statistical Analysis

In order to determine weekly patterns of search activity, we used our reconstructed time series to model the difference between Monday and the other days using day of the week as a factor variable (β_Tuesday_ + β_Wednesday_ + β_Thursday_ + β_Friday_ + β_Saturday_ + β_Sunday_) as described in other studies [20,21]. This allows us to determine the percentage increase in “sundowning” searches for each day of the week relative to the search activity on Mondays by the formula β_Day of the week_ / β_Intercept (Monday)_ * 100 [20,21]. As established in previous studies, confidence intervals were determined through bootstrap sampling of the ratio’s distribution (5000 simulations) [21,32].

The difference in search activity between winter (December, January, and February) and summer (June, July, and August) months was determined by a paired *t* test. The R core software package version 3.0.1 was used for statistical analysis with a significance level of *P*<.05 [30].

For our geographic pattern analysis, our primary response variable (Searches) was the normalized number of searches for the term “sundowning” on a state-by-state basis for 2016. Our predictor variables were the latitude of the center of each state (Latitude) and the percentage of time between sunrise and sunset that sunshine reaches the ground for each state (PercentSun), as used in previous investigations [24]. Density plots were visually inspected to identify data skewing. Any predictors that demonstrated skewing were logarithmically transformed (base 10) prior to the multivariable analyses [33]. Plots of residuals and a Q-Q (quantile-quantile) plot were examined for each model. For each simple linear regression, the coefficient of determination (*R*^2^), and beta coefficients (β) are reported [33]. The R core software package version 3.4.2 was used for statistical analysis with a significance level of *P*<.05 [30].

## Results

### Circumseptan Temporal Patterns

**Figure 1:**
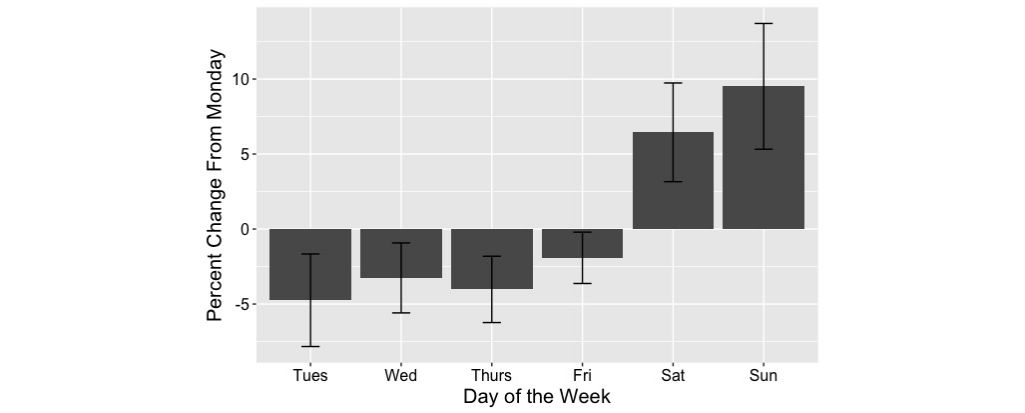
Circumseptan patterns of search activity in the United States for "sundowning" for each day of the week as compared to Monday.

None of our predictor or outcome variables demonstrated skewing on density plots. As shown in Figure 1, there was significant increase in search volume at the end of the weekend (Sunday) with searches being 9.5% (SD 4.2) higher. Sunday was the only significant factor variable, indicating that this was the only day of the week that showed a significant difference in search activity as compared to Monday. When all non-Sunday days were compared with Sunday searches, searches were 10.9% (SD 4.0) higher on Sunday as compared to the rest of the week. Google Trends normalizes search results for overall search activity to a score between 0 and 100.

### Seasonal Temporal Patterns

**Figure 2:**
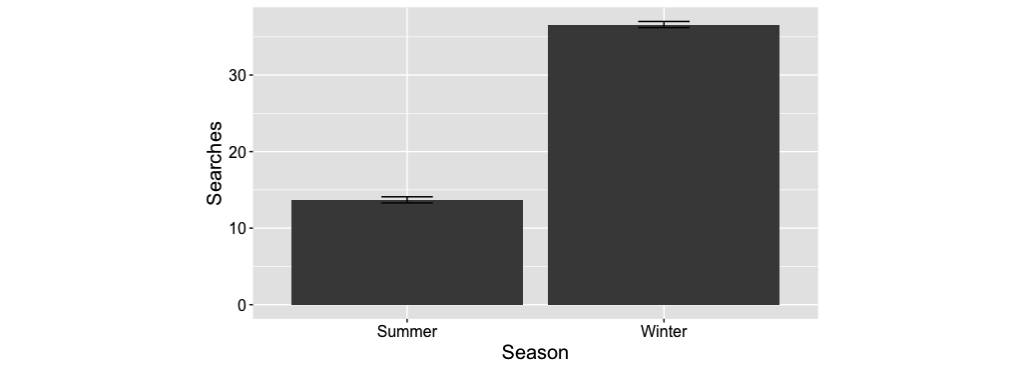
Seasonality of searches. Overall US search activity for "sundowning" for summer (June, July, and August) and winter (December, January, and February) months, showing much higher search activity during the winter.

Search activity showed a seasonal pattern with search activity significantly higher in the winter months while declining in the summer months (36.6 [SD 0.6] vs 13.7 [SD 0.2], *P*<.001) (Figure 2). Once again, Google Trends normalizes search results for overall search activity to a score between 0 and 100.

### State-by-State Variations by Sunshine Exposure and Latitude

**Figure 3:**
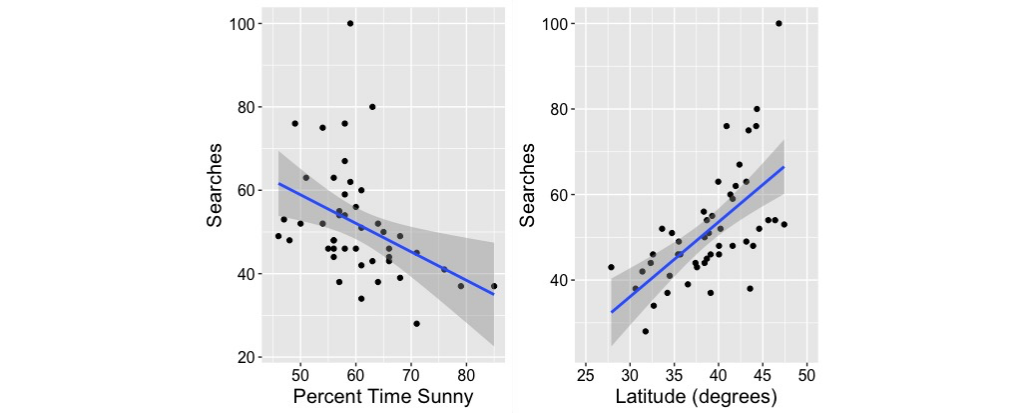
Search activity versus percent sunshine and latitude. Searches for "sundowning" showed a negative association with increasing percent daily sunshine and a positive association with increasing (more northerly) latitude.

State-by-state variations in “sundowning” searches showed a significant negative association with increasing PercentSun (*R*^2^=.16, β=-.429 [SD .149], *P*=.006), with states having a higher mean daily percent of number of sunny hours showing less search activity (Figure 3). Additionally, search activity was also higher in more northerly states, showing a positive association between sundowning searches and Latitude (*R*^2^=.38, β=.648 [SD .122], *P*<.001).

## Discussion

### Principal Findings

Health contemplations surrounding sundown syndrome in the United States showed both geographic patterns and temporal periodicities, specifically, (1) interest in sundown syndrome increased at the end of the weekend by approximately 10%, (2) search inquiries were much higher in winter as opposed to summer months, and (3) state-by-state search inquires showed a negative association with the average percent of the day that was sunny and a positive association with more northerly (increasing) latitudes.

The current study demonstrates an increase in search activity as the weekend progresses (Figure 1), with searches for “sundowning” approximately 10% higher on Sunday. Given the fact that our dataset reflects billions of individual Google searches [34], an increase of 10% would represent an increase on the scale of millions of health contemplations. Since Google Trends normalizes all search activity for overall general search activity, this indicates an increase in contemplations about sundown syndrome, not merely an increase in Internet use during the weekend. The suggestion that sundown syndrome is at least partially associated with caregiver fatigue as opposed to being solely due to changes in environmental light is supported by our results. We demonstrated an increase in “sundowning” searches at the end of every weekend, a time when families typically perform caregiving duties without any outside assistance [25]. The end of the weekend is in some respects the “end of a shift” for family caregivers until outside caregiving assistance returns with the start of the weekday.

Like most species, humans have endogenous circadian rhythms. Like all mammals, humans have a biological clock located in the superchiasmatic nuclei (SCN) in the hypothalamus that has both body temperature and melatonin as outputs [35]. The natural period of this rhythm is longer than 24 hours and requires synchronization via light information delivered from the retina to the SCN through the retinohypothalamic tract [36]. Both aging and neurodegenerative disease reduce the neuronal activity of the SCN [37], providing a potential biological basis for sundown syndrome behaviors.

Our study demonstrated an increased interest in searches for “sundowning” during winter months as opposed to summer months, in states that had a smaller percent of sunny days, and in more northerly states. Since Google Trends data are normalized for overall underlying search activity, this is not merely due to an increase in search activity during colder, more inclement weather. Previous work in human subjects has shown an inverse relationship between natural light exposure and the regularity of circadian rhythms. In fact, the seasonal reduction in natural light exposure during the winter months has been linked with increased disruption of circadian rhythms [38]. Our results suggest that the reduction in natural light exposure during the winter months is one potential explanation for sundown syndrome behaviors. Caregiver stress cannot logically be the only explanation for this phenomenon. Amyloid precursor protein mice models show sundown syndrome behaviors similar to that described clinically [7] and there is certainly no “caregiver stress” in this scenario. As well, studies of light therapy [11], melatonin [14], and increased exposure to natural light [13] in cognitively impaired persons living in facilities all support disrupted circadian rhythms as a contributing factor to sundown syndrome behavior.

### Clinical Implications

Our analysis of Google Trends data has demonstrated that health contemplations about sundown syndrome are higher at the end of the weekend, higher in winter months, and higher in states with less sunshine/more northerly latitudes. This ability to examine people’s hidden contemplations could potentially allow us to target home supports more effectively. As an example, more resources for home supports could be deployed during the winter months or in portions of the country that get less natural light. The downsides of reduced person-power on weekends has been well demonstrated in the acute care setting [39], suggesting that these same issues may be increasing caregiver stress in the community setting as well. Our study provided correlational evidence for both the caregiver stress hypotheses and the circadian rhythm hypothesis for sundown syndrome.

### Limitations

Although our study is suggestive with respect to the weekly, seasonal, and geographic patterns of healthy contemplations surrounding “sundowning,” Google search activity does not indicate the underlying context for each search. Further research needs to be done to determine if targeting more home supports on the weekend, the winter months, and less sunny geographic locations would be a more efficient way to deploy health care resources.

In addition, Google Trends provides only normalized results of search data as opposed to absolute numbers of searches. Offsetting this, however, is the fact that the number of keyword searches are in the billions [40], so any observed seasonal, weekly, or geographic increase in normalized results likely represent millions of additional searches. For our search terms, we chose the colloquially used term “sundowning” as opposed to other more clinical search terms (eg, “sundown syndrome”) in order to better target the layperson population, which is a potential limitation.

Our study also considered searches only in the United States. National differences due to differences in health care systems might have conceivably changed search behavior and is a potential future avenue of research. We also examined searches only in the “Health” section of Google trends, which might have omitted search queries related to caregiving and topics covered by the social science literature.

### Conclusions

Health contemplations surrounding sundown syndrome behaviors are higher at the end of weekends, in less sunny states, in states at higher latitudes, and during winter months. These results provide support for both the caregiver stress and disrupted circadian rhythm hypotheses for sundown syndrome behaviors.

## Abbreviations

SCN: superchiasmatic nuclei

## References

[1] Beeri MS, Werner P, Davidson M, Noy S (2002). The cost of behavioral and psychological symptoms of dementia (BPSD) in community dwelling Alzheimer's disease patients. Int J Geriatr Psychiatry.

[2] Bitwise D (1994). What is sundowning?. J Am Geriatr Soc.

[3] Gallagher-Thompson D, Brooks JO, Bliwise D, Leader J, Yesavage JA (2015). The Relations among Caregiver Stress, “Sundowning” Symptoms, and Cognitive Decline in Alzheimer's Disease. J Am Geriatr Soc.

[4] Cameron DE (1941). Studies in senile nocturnal delirium. Psych Quar.

[5] Evans LK (1987). Sundown syndrome in institutionalized elderly. J Am Geriatr Soc.

[6] Exum ME, Phelps BJ, Nabers KE, Osborne JG (1993). Sundown syndrome: is it reflected in the use of PRN medications for nursing home residents?. Gerontologist.

[7] Bedrosian TA, Herring KL, Weil ZM, Nelson RJ (2011). Altered temporal patterns of anxiety in aged and amyloid precursor protein (APP) transgenic mice. Proc Natl Acad Sci U S A.

[8] Bliwise DL, Lee KA (1993). Development of an Agitated Behavior Rating Scale for discrete temporal observations. J Nurs Meas.

[9] Figueiro MG (2017). Light, sleep and circadian rhythms in older adults with Alzheimer's disease and related dementias. Neurodegener Dis Manag.

[10] Figueiro MG, Hamner R, Higgins P, Hornick T, Rea MS (2012). Field measurements of light exposures and circadian disruption in two populations of older adults. J Alzheimers Dis.

[11] Wahnschaffe A, Nowozin C, Haedel S, Rath A, Appelhof S, Münch M, Kunz D (2017). Implementation of Dynamic Lighting in a Nursing Home: Impact on Agitation but not on Rest-Activity Patterns. Curr Alzheimer Res.

[12] Fontana Gasio P (2003). Dawn–dusk simulation light therapy of disturbed circadian rest–activity cycles in demented elderly. Experimental Gerontology.

[13] Gammack JK, Burke JM (2009). Natural light exposure improves subjective sleep quality in nursing home residents. J Am Med Dir Assoc.

[14] Tampi RR, Tampi DJ, Balachandran S (2017). Antipsychotics, Antidepressants, Anticonvulsants, Melatonin, and Benzodiazepines for Behavioral and Psychological Symptoms of Dementia: a Systematic Review of Meta-analyses. Curr Treat Options Psych.

[15] Cohen-Mansfield J (1986). Agitated behaviors in the elderly. II. Preliminary results in the cognitively deteriorated. J Am Geriatr Soc.

[16] Rimmer E, Wojciechowska M, Stave C, Sganga A, O'Connell B (2005). Implications of the Facing Dementia Survey for the general population, patients and caregivers across Europe. Int J Clin Pract Suppl.

[17] Ayers JW, Althouse BM, Dredze M (2014). Could behavioral medicine lead the web data revolution?. JAMA.

[18] van de Mortel TF (2008). Faking It: Social Desirability Response Bias in Self-report Research. Aust J Adv Nurs.

[19] Eysenbach G (2011). Infodemiology and infoveillance tracking online health information and cyberbehavior for public health. Am J Prev Med.

[20] Ayers JW, Althouse BM, Johnson M, Cohen JE (2014). Circaseptan (weekly) rhythms in smoking cessation considerations. JAMA Intern Med.

[21] Ayers JW, Althouse BM, Johnson M, Dredze M, Cohen JE (2014). What's the healthiest day?: Circaseptan (weekly) rhythms in healthy considerations. Am J Prev Med.

[22] Madden KM (2017). The Seasonal Periodicity of Healthy Contemplations About Exercise and Weight Loss: Ecological Correlational Study. JMIR Public Health Surveill.

[23] Ingram DG, Plante DT (2013). Seasonal trends in restless legs symptomatology: evidence from Internet search query data. Sleep Med.

[24] Ayers JW, Althouse BM, Allem J-P, Rosenquist JN, Ford DE (2013). Seasonality in seeking mental health information on Google. Am J Prev Med.

[25] Ward-Griffin C, Hall J, Deforge R, St-Amant O, McWilliam C, Oudshoorn A, Forbes D, Klosek M (2012). Dementia home care resources: how are we managing?. J Aging Res.

[26] (2015). Google.

[27] Nuti SV, Wayda B, Ranasinghe I, Wang S, Dreyer RP, Chen SI, Murugiah K (2014). The use of google trends in health care research: a systematic review. PLoS One.

[28] (2019). Office of Research Ethics.

[29] Schmidbauer H, Roesch A (2018). WaveletComp: A guided tour through the R-package.

[30] R Foundation for Statistical Computing (2017). R Core Team.

[31] (2017). National Centers for Environmental Information.

[32] Davison AC (1997). Bootstrap Methods And Their Application (Cambridge Series In Statistical And Probabilistic Mathematics).

[33] Chatterjee S, Hadi AS (2006). Regression Analysis by Example, 4th Ed.

[34] Eysenbach G (2009). Infodemiology and infoveillance: framework for an emerging set of public health informatics methods to analyze search, communication and publication behavior on the Internet. J Med Internet Res.

[35] Moore RY (1997). Circadian rhythms: basic neurobiology and clinical applications. Annu Rev Med.

[36] Wever RA, Polásek J, Wildgruber CM (1983). Bright light affects human circadian rhythms. Pflugers Arch.

[37] Swaab DF, Fliers E, Partiman TS (1985). The suprachiasmatic nucleus of the human brain in relation to sex, age and senile dementia. Brain Res.

[38] Lewy AJ, Lefler BJ, Emens JS, Bauer VK (2006). The circadian basis of winter depression. Proc Natl Acad Sci U S A.

[39] Concha OP, Gallego B, Hillman K, Delaney GP, Coiera E (2014). Do variations in hospital mortality patterns after weekend admission reflect reduced quality of care or different patient cohorts? A population-based study. BMJ Qual Saf.

[40] Houston TK, Volkman JE, Feng H, Nazi KM, Shimada SL, Fox S (2013). Veteran internet use and engagement with health information online. Mil Med.

